# Rapid Artificial Intelligence Solutions in a Pandemic - The COVID-19-20 Lung CT Lesion Segmentation Challenge

**DOI:** 10.21203/rs.3.rs-571332/v1

**Published:** 2021-06-04

**Authors:** Holger R. Roth, Ziyue Xu, Carlos Tor Diez, Ramon Sanchez Jacob, Jonathan Zember, Jose Molto, Wenqi Li, Sheng Xu, Baris Turkbey, Evrim Turkbey, Dong Yang, Ahmed Harouni, Nicola Rieke, Shishuai Hu, Fabian Isensee, Claire Tang, Qinji Yu, Jan Sölter, Tong Zheng, Vitali Liauchuk, Ziqi Zhou, Jan Hendrik Moltz, Bruno Oliveira, Yong Xia, Klaus H. Maier-Hein, Qikai Li, Andreas Husch, Luyang Zhang, Vassili Kovalev, Li Kang, Alessa Hering, João L. Vilaça, Mona Flores, Daguang Xu, Bradford Wood, Marius George Linguraru

**Affiliations:** 1 NVIDIA, Bethesda, MD, USA; 2 Sheikh Zayed Institute for Pediatric Surgical Innovation, Children’s National Hospital, Washington, DC, USA; 3 Division of Diagnostic Imaging and Radiology, Children’s National Hospital, Washington, DC, USA; 4 Radiology and Imaging Sciences, National Institutes of Health Clinical Center, Bethesda, MD, USA; 5 School of Computer Science and Engineering, Northwestern Polytechnical University, China; 6 HIP Applied Computer Vision Lab, German Cancer Research Center (DKFZ), Heidelberg, Germany; 7 Lynbrook High School, San Jose, CA, USA; 8 Shanghai Jiao Tong University, China; 9 Luxembourg Centre for Systems Biomedicine, University of Luxembourg, Luxembourg; 10 School of Informatics, Nagoya University, Japan; 11 Biomedical Image Analysis Department, United Institute of Informatics Problems, Belarus; 12 Guangdong Key Laboratory of Intelligent Information Processing, Shenzhen University, China; 13 Fraunhofer Institute for Digital Medicine MEVIS, Bremen, Germany; 14 Life and Health Sciences Research Institute (ICVS), School of Medicine, University of Minho, Braga, Portugal; 15 ICVS/3B’s - PT Government Associate Laboratory, Braga/Guimarães, Portugal; 16 Algoritmi Center, School of Engineering, University of Minho, Guimarães, Portugal; 17 School of Computer Science and Engineering, Northwestern Polytechnical University, China; 18 Division of Medical Image Computing, German Cancer Research Center (DKFZ), Heidelberg, Germany; 19 Fraunhofer Institute for Digital Medicine MEVIS, Lübeck, Germany; 20 2Ai – Polytechnic Institute of Cávado and Ave, Barcelos, Portugal; 21 School of Medicine and Health Sciences, George Washington University, Washington, DC, USA

**Keywords:** COVID-19, artificial intelligence, chest computed tomography

## Abstract

Artificial intelligence (AI) methods for the automatic detection and quantification of COVID-19 lesions in chest computed tomography (CT) might play an important role in the monitoring and management of the disease. We organized an international challenge and competition for the development and comparison of AI algorithms for this task, which we supported with public data and state-of-the-art benchmark methods. Board Certified Radiologists annotated 295 public images from two sources (A and B) for algorithms training (n=199, source A), validation (n=50, source A) and testing (n=23, source A; n=23, source B). There were 1,096 registered teams of which 225 and 98 completed the validation and testing phases, respectively. The challenge showed that AI models could be rapidly designed by diverse teams with the potential to measure disease or facilitate timely and patient-specific interventions. This paper provides an overview and the major outcomes of the COVID-19 Lung CT Lesion Segmentation Challenge - 2020.

## Introduction

The SARS-CoV-2 pandemic has had a devastating impact on the global healthcare systems. As of May 28, 2021, more than 169 million people have been infected in the world with over 3.5 million deaths ^1^. COVID-19 is known to affect nearly every organ system, including the lungs, brain, kidneys, liver, gastrointestinal tract, and cardiovascular system. The manifestations of the disease in the lung may be early indicators of future problems. These manifestations have been intensively reported in the adult populations and occasionally in pediatric subjects ^2–6^. Since the early days of the pandemic, lung imaging has been critical for both the early identification and management of individuals affected by COVID-19 ^7^. Imaging also provides invaluable support for the evaluation of patients with long COVID and after the acute sequelae of the diseases. Repeated waves of infection and changes in the disease course require data, including imaging, classification, quantification, and response tools, as well as standardized reliable interpretation as the global society struggles to provide widely available vaccines and faces evolving challenges such as new mutations of the virus.

The most common lung imaging modalities utilized for the evaluation of SARS-CoV-2 infections are chest radiographs (CXR) and chest computerized tomography (CT) with ultrasound (US) being used more sparingly. Chest CT is the reference modality that most accurately demonstrates the acute lung manifestations of COVID-19 ^8,9^. As observed in CT, the most common findings in the chest of the affected individuals were ground-glass opacities (GGO) and pneumonic consolidations. Other manifestations include interstitial abnormalities, crazy paving pattern, halo signs, pleural abnormalities, bronchiectasis, bronchovascular bundle thickening, air bronchograms, lymphadenopathy, and pleural/pericardial effusions. The sensitivity of chest CT to detect these abnormalities in subjects with confirmed COVID-19 was widely variable and somewhat subjective, reported in the range of 44–97% (median 69%) ^10^.

Beside its role in the identification of patterns of SARS-CoV-2 infections, lung CT is also important in the determination of the severity of COVID-19 ^6,8,9,11,12^. The presence, location and extension of the lung abnormalities are critical factors for the clinical management of patients to potentially facilitate decisions towards more timely and personalized medical interventions. Quantification of lesions may further provide the tracking of disease progression and response to therapeutic countermeasures. Thus, improving COVID-19 treatment starts with a clearer understanding of the patient’s disease state, which must include accurate identification, delineation and quantification of lung lesions and disease phenotypes and patterns.

A prior lack of global data collaboration limited clinicians and scientists in their ability to quickly and effectively understand COVID-19 disease, its severity and outcomes. As access to data has improved, quality annotations have remained a limiting factor in the development of useful artificial intelligence (AI) models derived from machine learning and deep learning ^13^. Thus, a multitude of AI approaches have been developed, published and indicated great potential for clinical support, but they were often overfit, being trained using proprietary data or from a single site ^14–19^. Alternatively, federated approaches allow algorithms to access data from multiple sites without the need of sharing raw data, but through this paradigm access is granted to a single algorithm and consortium, with sharing of model weights instead of raw data ^20,21^. In particular, deep neural networks were used for the identification and segmentation of abnormal lung regions affected by SARS-CoV-2 infection. These can be grouped into two main classes: classification models that extract the affected region inside the lung area by comparison with data from healthy subjects ^22–25^, and segmentation models that directly extract the abnormal lung areas according to patterns in the image and (typically using fully convolutional networks) ^16,18,26–28^.

Without access to public data and an adequate platform to evaluate and compare their performance, AI approaches risk being overtrained, irreproducible, and ultimately clinically not useful. Thus, public efforts are needed to accelerate the understanding of the role of AI towards informing manifestations and qualifying impact of health crises such as the COVID-19 pandemic.

The COVID-19 Lung CT Lesion Segmentation Challenge 2020 (COVID-19–20) created the public platform to evaluate emerging AI methods for the segmentation and quantification of lung lesions caused by SARS-CoV-2 infection from CT images. This effort required a multi-disciplinary team science partnership among global communities in a broad variety of often disparate fields, including radiology, computer science, data science and image processing. The goal was to rapidly team up to combine multi-disciplinary expertise towards the development of tools to simultaneously both define and address unmet clinical needs created by the pandemic. The COVID-19–20 platform provided access to multi-institutional, multinational images originating from patients of different ages and gender, and with variable disease severity. The challenge team provided the ability to quickly label a public dataset, allowing radiologists to rapidly add precise annotations. Open access was offered to the annotated CTs of subjects with PCR-confirmed COVID-19, and to a baseline deep learning pipeline based on MONAI ^29^ that could serve as a starting point for further algorithmic improvements. The challenge was hosted on a widely used competition website (covid-segmentation.grand-challenge.org) for easy and secure data access control. This paper presents an overview of this challenge and competition, including the data resources and the top ten AI algorithms identified from a highly competitive field of participants who tested the data in December 2020.

### Submissions

**Figure 1 |:**
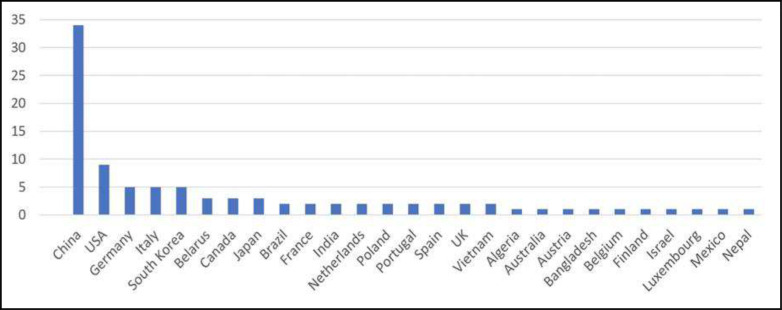
The countries of origin of the 98 teams that completed the training, validation and test phases of the challenge.

**Figure 2 |:**
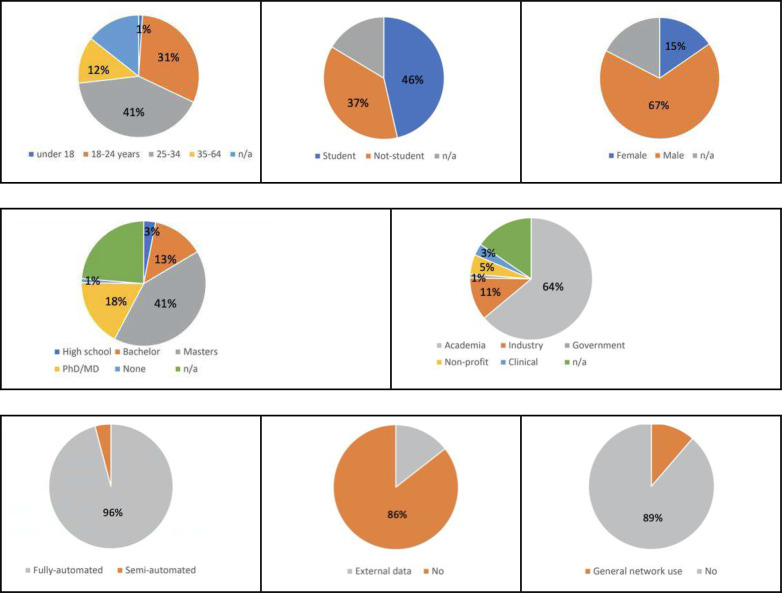
Demographic information of the leaders of the 98 teams that completed the training, validation and test phases of the challenge. The top row shows the age group (left), student status (middle) and sex (right) of the participant. The middle row shows the highest degree (left) and job category (right). Bottom row shows the algorithm characteristics for the 98 submissions that completed the training, validation and test phases of the challenge. We report if algorithms were fully-automated (left), used external data for training (middle) or used a general pre-trained network for initialization (right).

The challenge was launched on November 2, 2020. The training and validation data were released and 1,096 teams registered before the training phase was closed on December 8, 2020. The 225 teams that completed the validation phase were given access to the test data. Ninety-eight teams from 29 countries on six continents completed the test phase. Figure 1 shows the countries of origin of the 98 teams. Test results were released on December 18, 2020, and the statistical ranking of the top ten teams (see Results) was unveiled during a virtual mini symposium on January 11, 2021 ^30^. Figure 2 shows the demographic information for the team leaders, i.e., age group, sex, highest educational degree, student status and job category, and algorithmic characteristics for the 98 submissions that completed the training, validation and test phases. We requested participants to disclose whether they used external data for training their algorithms or if they used a general-purpose pre-trained network for initialization (e.g., a network pre-trained for another lung disease). The use of public networks pre-trained for the segmentation of COVID-19 lesions was not allowed (e.g., Clara_train_covid19_ct_lesion_seg ^31^).

Participants uploaded the results on the validation and test data to the hosting website for evaluation. Only (semi-)automated methods were allowed. Submission of manual annotations was prohibited. For validation, the number of submissions from each user was limited to once-a-day for the purpose of refining their algorithms based on the live performance indicators on the challenge validation leaderboard ^32^. Submission of results on the test data was collected without showing the leaderboard and the last submission was used for final ranking. The test phase was open only to participants who had already submitted their results on the validation set. The leaderboard and final ranking are public and hosted on the challenge website ^33^.

## Results

### Data sources

This challenge utilized data from two public resources on chest CT images, namely the “CT Images in COVID-19” ^34,35^ (Dataset 1) and “COVID-19-AR”^36^ (Dataset 2) available on The Cancer Imaging Archive (TCIA) ^36^. CT images were acquired without intravenous contrast enhancement from patients with positive Reverse Transcription Polymerase Chain Reaction (RT-PCR) for SARS-CoV-2. Dataset 1 originated from China, while dataset 2 was acquired from the US population. In total, we used 295 images, including 272 images from Dataset 1 and 23 images from Dataset 2. Of these images, 199 and 50 from Dataset 1 were used for training and validation, respectively. We therefore refer to Dataset 1 as the “seen” data source that participants used to train and validate their algorithms during the first phase of the challenge. The test set contained 23 images each from Datasets 1 and 2 (46 images in total). Hence, Dataset 2 was only used in the testing phase, and we refer to it as the “unseen” data source.

**Figure 3 |:**
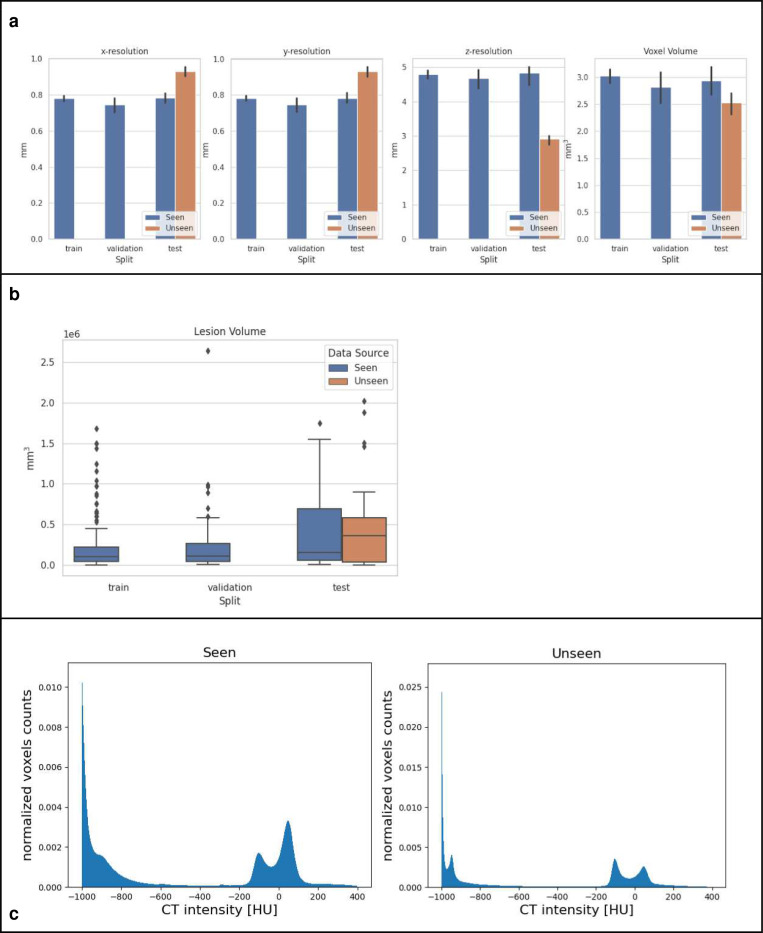
Data variability between “seen” and “unseen” sources; a) Illustration of the differences in the image resolution and voxel volume grouped by training, validation, and test sets. b) Differences in COVID-19 lesion volumes across the image data sources. c) Normalized histograms showing the CT intensity distributions of the “seen” and “unseen” data sources in Hounsfield units (HU). Note, −1000 HU corresponds to air, and 750 to cancellous bone ^37^.

Descriptive statistics, such as x-, y-, and z-resolutions and voxel volume in both data sources are shown in Figure 3. We also show the differences in COVID-19 lesion volumes annotated between the two data sources.

### Annotation protocol

All images were automatically segmented by a previously trained model for COVID lesion segmentation^20^ that is publicly available ^38^. All lung lesions related to COVID-19 were included. These segmentations were subsequently used as a starting point for board certified radiologists (RS, JZ, JM) who manually adjudicated and corrected them. The annotation tool used was ITKSnap^39,40^ showing multiple reformatted views of the CT scans, and allowing manipulations and corrections of the initial automated segmentation results in three dimensions.

### Evaluation metrics

We used the three evaluation metrics described below. These metrics were both used to evaluate the performance of different algorithms, and to establish the interobserver variability.

1. *Dice Coefficient (Dice).* A common evaluation metric of segmentation accuracy defined as the overlap between the volume of the ground truth segmentation *S*_*gt*_ and the predicted segmentation volume *S*_*pred*_; $\mathrm{Dice}=\frac{2\times \left(S_{gt}\;\cap \;S_{\mathrm{pred}}\right)}{S_{\mathrm{gt}}\;\cup \;S_{\mathrm{pred}}}.$
2. *Normalized Surface Dice (NSD).* Similarly to Dice, it provides the normalized measure of agreement between the surface of the prediction and the surface of the ground truth^41^. We chose a threshold of 1mm to define an “acceptable” derivation between the ground truth surface and the predicted surface.
3. *Normalized Absolute Volume Error (NAVE).* The volume of COVID-19 lesion burden inside the patient’s lung can play an important role for clinical assessment ^42^. Therefore, a measure was chosen that assesses the agreement between the predicted and ground truth lesion volumes, defined as $V_{\mathrm{error}}=\frac{\left|V_{\mathrm{pred}}-V_{gt}\right|}{V_{gt}}$. Note, we used the negative of this value for ranking purposes as higher values indicate better performance in our ranking approach.

### Interobserver performance

As a benchmark for comparing the AI algorithms with human performance on the lesion segmentation task, we measured the human interobserver agreement. We compared the annotations utilized in Yang et al.^20^ from 245 of the 272 cases from Dataset 1 used in the challenge with the ones obtained by our radiologists. The interobserver agreement showed mean ± standard deviation (median) of Dice, NSD, and NAVE of 0.702 ± 0.172 (0.756), 0.538 ± 0.147 (0.563), and 0.601 ± 1.969 (0.180), respectively.

### Statistical ranking method

Recent work on ranking analysis for biomedical imaging challenges has shown that ranking results can vary significantly depending on the chosen type of metric and ranking scheme ^43^. Most biomedical challenges use approaches such as “aggregate-then-rank” or “rank-then-aggregate”, which do not account for statistical differences between algorithms ^43,44^. These findings motivated the development of a challenge ranking toolkit ^44,45^ that we employed for our evaluation. This toolkit utilizes statistical hypothesis testing applied to each possible pair of algorithms. This allows us to better assess the differences between the evaluated metrics.

**Figure 4 |:**
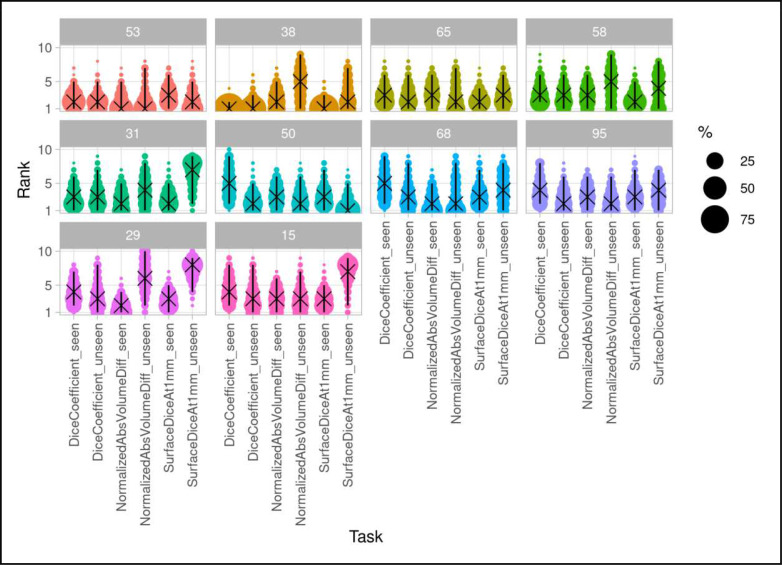
Blob plot visualization of the ranking variability via bootstrapping. An algorithm’s ranking stability is shown across the different tasks, illustrating the ranking uncertainty of the algorithm in each task. For more details see ^44^.

Following the notation of Wiesenfarth et al ^44^, our challenge contained *m* = 6 tasks (Dice, NSD, NAVE on each “seen” and “unseen” test data). The test cases for each task are denoted as *n*_*k*_, *k* = 1, . . . , *m*_*test*_. In our case, *m*_*test*_ = 23for each task. A bootstrap approach is used to evaluate the ranking stability of the different algorithms. This means that ranking is performed repeatable on *b*= 1,000bootstrap samples, see Figure 4. The statistical test employed to determine the consensus ranking is the one-sided Wilcoxon signed rank test with a significance level of *α* = 5%, adjusted for multiple testing according to Holm ^44^.

Each of the *m* tasks contributed equality to the final consensus using the Euclidean distance between averaged ranks across tasks. We ranked the 98 submitted algorithms using the proposed statistical consensus ranking algorithm to determine the top-10 methods, including the challenge winning algorithm.

### Summary of top-10 algorithms

We show the final ranking of the top-10 performing algorithms in Table 1. All top-10 algorithms were fully-automated methods, and all were based on some variation of the U-Net^46,47^, a fully convolutional network^48^ for image segmentation based on the popular encoder-decoder design with skip connections ^48,49^. U-Net has dominated the field of biomedical image segmentation in recent years ^50^ and most challenge participants opted to use one of its implementations. In particular the nnU-Net open-source framework ^51,52^, which has shown success in multiple biomedical image segmentation challenges, was a popular choice for challenge participants. The U-Net architectures included 2D, 3D, high and low resolution configurations. One team used the open-source platform MONAI ^53^ (#68). The majority of algorithms used challenge data only with one method including additional unlabeled data from the public TCIA source (#53), which was done with pseudo labels in a semi-supervised approach. The majority directly targeted the segmentation of COVID-19 lesions, while one participant (#31) targeted multiple outputs, including body and lung masks.

A popular loss function for biomedical image segmentation is the Dice loss ^54^. In this challenge, most finalists utilized it together with additional cross entropy, top-k ^55^, and focal loss ^56^. An important strategy for winning image segmentation is model ensembling, the fusion of predictions from several independently trained models. Here, most methods used 5-fold cross validation and model ensemble to arrive at a consensus prediction.

A full description of the top-10 finalists’ algorithms by their authors is given in Methods.

### Ranking results

Table 2 shows the mean and standard deviation of the Dice coefficients for the top-10 performing algorithms on test cases from the “seen” and “unseen” data sources. Top algorithms performed relatively similar to each other, but all showed a marked decrease when being evaluated on the “unseen” data (Table 2).

**Figure 5 |:**
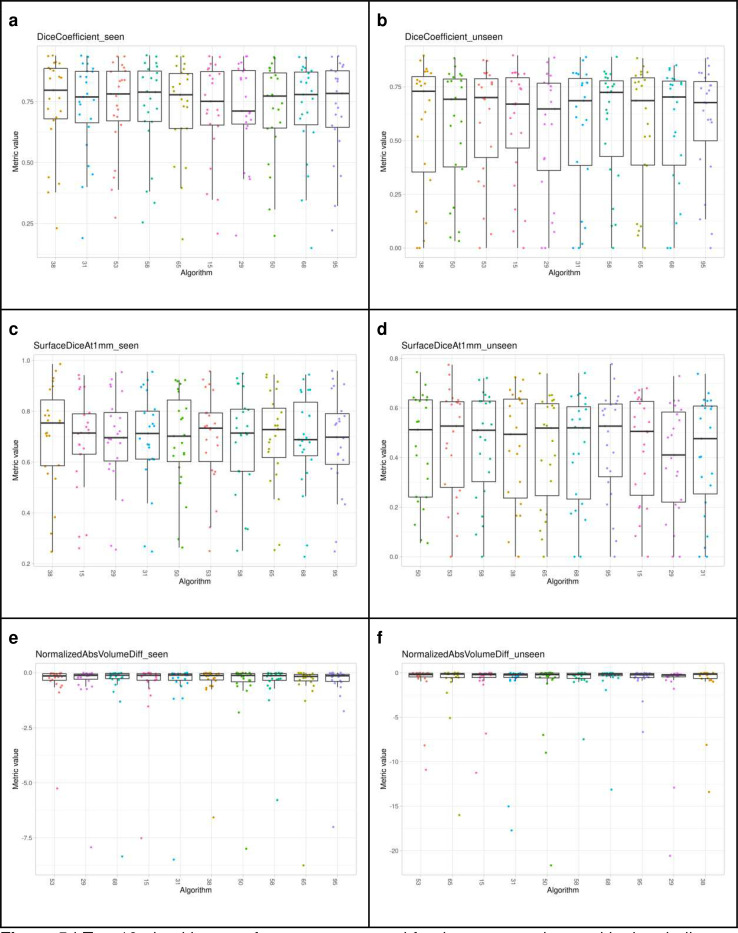
Top-10 algorithms performance measured for the *m* = 6 tasks used in the challenge, namely the Dice coefficient (top row), Normalized Surface Dice (middle row), and Normalized Absolute Volume Error (bottom row) on the “seen” (a, c, e) and “unseen” test datasets (b, d, f), respectively. Algorithms are ranked based on their performance from left to right.

**Figure 6 |:**
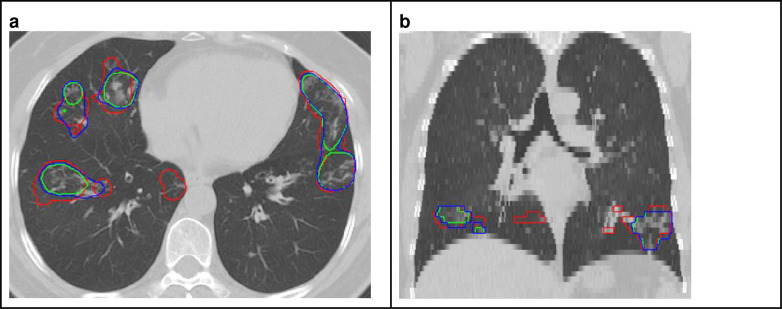
Example test case from the “seen” data data source (Dataset 1). The performance of the top algorithms #53 and #38 is shown in green and blue, respectively. Ground truth annotations are shown in red (a: axial view, b: coronal view).

**Figure 7 |:**
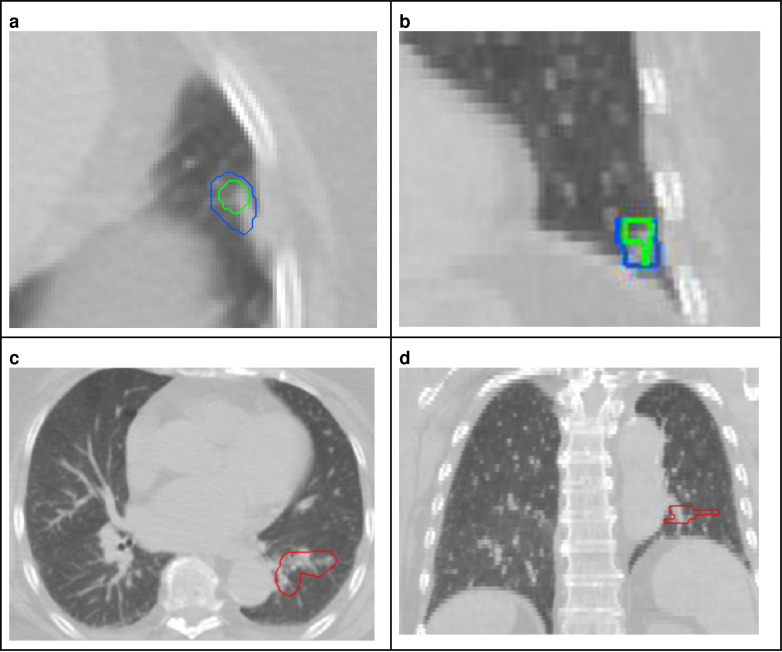
Example test case from the “unseen” data source. (a: axial view, b: coronal view) Top algorithms #53 and #38, shown in green and blue, respectively, both predict a false-positive lesion at the locations of a normal lung vessel. At the same time they missed the real lesion in red (c: axial view, d: coronal view).

**Figure 8 |:**
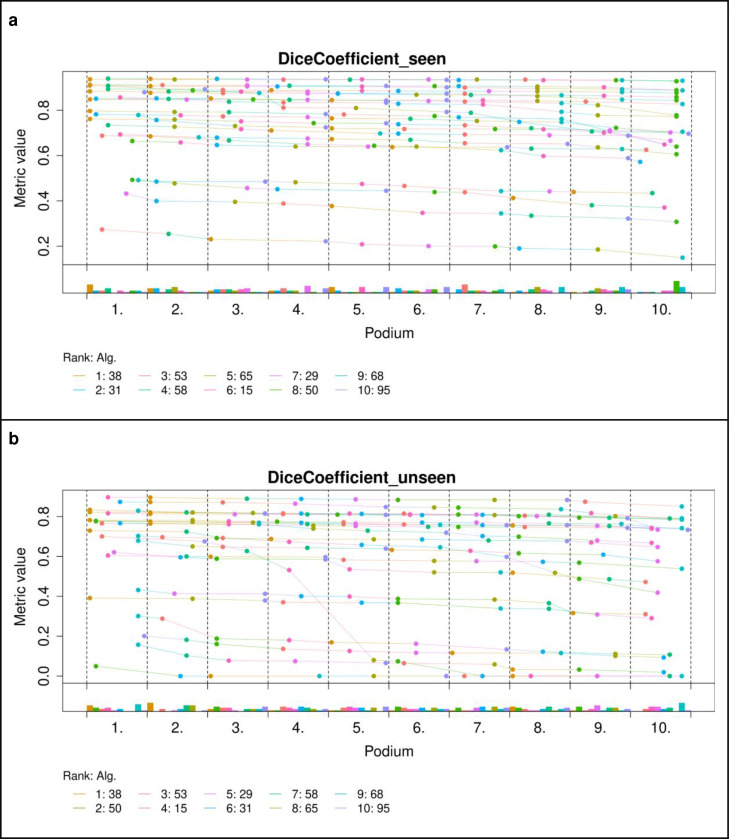
Podium plots for “seen” (a) and “unseen” (b) test data. The participating algorithms are color-coded. Each colored dot shows the Dice coefficient achieved by the respective algorithm. The same test cases are connected by a line. The lower part of the charts displays the relative frequency for a given algorithm to achieve a podium place, i.e. rank achieved by a given algorithm.

Figure 5 shows boxplots of the top-10 performing algorithms for each of the *m* = 6tasks. In general, methods present more outliers on the “unseen” test dataset. Figure 6 shows a typical example from the “seen” test data source. The top-performing algorithms (#53 and #38) achieved a mean Dice coefficient >0.734 Dice on the “seen” dataset. Figure 6 shows that most of the COVID-19 related lesions were well segmented by the automated algorithms. In contrast, Figure 7 shows a challenging case from the “unseen” test data source. Both top-performing algorithms (#53 and #38) generated a false-positive segmentation region at a normal lung vessel while missing the real lesion. Their performance dropped to a Dice coefficient <0.598 on the “unseen” dataset. To illustrate the general performance of the top-10 algorithms on the individual test cases, Figure 8 shows podium plots ^57^ with the performance of different algorithms on the same test case connected by a line.

## Discussion

### Performance of algorithms

Automatic AI algorithms showed great potential to accurately segment the lung COVID-19 lesions from CT images. In the validation phase, 87 out of 225 methods achieved superior Dice coefficients than the interobserver criteria (0.702), with the top team achieving a Dice coefficient of 0.771 (~9.8% improvement). However, their level of robustness is inferior to the radiologist’s performance: the top team gets a Dice coefficient of 0.666 on the test data ^58^ (~5.1% decrease). This discrepancy could be due to various reasons. One reason could be the domain shift as half of the test data is from an “unseen” source that has not been used in the training or validation phases. Another reason could be the limited number of allowed submissions for the testing phase, which mitigates the possibility for overfitting to the test data. Moreover, the limited number of training data could also affect algorithm performance.

The evaluation of the analysis of top-10 algorithms revealed that the ensemble of segmentation from various individual automated methods plays an important role compared to other factors such as the complexity of the network architecture, the learning rate, losses, etc. Most 10 top teams used model ensembles to reduce outliers and improved their performance by collecting the consensus segmentation from separately trained models. This observation also shows that the training pipeline can potentially be further improved based on novel concepts like AutoML ^59,60^ or neural architectures search ^61–63^ algorithms.

### Use of external training data

Only one of the top 10 teams, which was the winning team of the challenge, used external data in their final solution. Using this semi-supervised training approach, they obtained an improvement of 4.27% and 0.86% Dice coefficient on the training and validation data, respectively. Another team did similar work in a student-teacher manner and saw improvement in the validation score. However, they submitted their final results without using the external data after noticing partial overlap between the chosen unlabelled external dataset and the provided training data. Both teams demonstrate that using external data, even unlabelled, could improve the segmentation performance. While this finding clearly calls for larger training datasets, it also shows the great potential of semi-supervised methods to achieve more robust solutions, especially for the healthcare domain where the annotation cost is much higher than in other fields ^64^.

### U-Net dominance

All top-10 teams used a 2D/3D U-Net variant with at most minor modifications. While this seems to conflict with hundreds of yearly publications creating new network architectures, it also shows that most existing deep learning algorithms lack the robustness offered by model ensembles to handle large data variations (e.g., resolution, contrast, etc.) when training data are limited. nnU-Net ^51^ was adopted by 5 out of the 10 teams to build an end-to-end solution while another team used MONAI ^65^. Unsurprisingly, these findings show that the majority of participants employed well-validated, open-source resources.

### Data variability and generalizability gap

The challenge was designed to use “seen” and “unseen” data sources and thus evaluate the generalizability of AI algorithms in front of variable clinical protocols. Our data sources varied in provenience (China and US), scanner manufacturers (various, as typical in routine clinical practice) and imaging protocols (image resolution). Figure 3 illustrates that the volumes of the annotated COVID lesions have similar distributions on the two data sources. However, there are substantial differences in the image resolution used for CT reconstruction in the data. These differences in voxel resolution, together with variability in scanner manufacturers and imaging protocols, were likely the main contributors to the generalization gap seen in the performance of algorithms on the “unseen” test cases. Additional factors were related to the variability of manifestations of the disease in the lungs. For examples, in the challenging case from the “unseen” test data source shows in Figure 7, the top-performing algorithms generated false-positive predictions at a normal lung vessel while missing to segment the real lesion. Domain shifts like the ones observed in the data used in this challenge are still proving to be challenging for current AI models. Disease phase variability may also have broadened the features of what defines a standard or expected set of features. Early disease may not look like later disease cycles on CT, which may have also increased model noise.

### Potential for clinical use

Segmentation and classification models have been postulated to impact diagnosis in outbreak settings with delayed or unavailable PCR, however the point of care classification of COVID-19 versus other pneumonia such as influenzae, could prove of some value during flu season in specific outbreak settings as an epidemiologic tool or as a red flag for patient isolation at the scanner, by early identification, thus expediting or prioritizing interpretation using more conventional radiologist review and verification. AI models have also been proposed to assist in triage or selection of resource-limited therapeutics or critical care, prognostication or prediction of outcomes, or as one data element of a multi-modal model combining clinical, laboratory and imaging data. Standardized response criteria for clinical trials can provide a level “playing field”, thus uniformly defining effects of medical and other countermeasures, or specific scenarios for patient-specific therapies. Specific phenotypes may respond to certain therapies, for example. Imaging AI could thus play a role in determining the optimal disease phase for steroid administration or monoclonal antibodies, or even characterize the presence of different disease manifestations according to variant or underlying comorbidity, although many of these clinical or research utilities are quite speculative. AI models in COVID-19 have been justifiably criticized for a lack of generalize-ability, lack of clinical testing and validation, impracticality of model design, “me-too” models and studies, and easy replaceability of functionality with standard clinical tools. Potential clinical impact has yet to match the excitement from the data science and computational community nor realize the promise at the outset of the pandemic. Federated learning and open-source tools and modeling may help address this, especially for specific research questions for clinical trials or radiologist-sparse settings.

### Limitations

The challenge organizers aimed to create a fair and robust evaluation platform for (semi)automatic AI algorithms. This was a timely effort completed with limited resources, thus several factors could potentially be improved in retrospect. For example, 295 annotated CT images from two different data sources were used in the challenge, which may be suboptimal data quantity for training deep learning algorithms, as performance metrics improve with size of datasets. However, the challenge set a benchmark for the development and evaluation of AI methods to segment lung lesions in COVID-19, the first of its kind to our knowledge, which was reflected by the large number of participants. It is advisable to add more data in future challenges, even if the data are non-annotated as the results of this challenge indicated.

Another limitation may be the data annotation. Each case was annotated by one radiologist who rectified the prediction from a publicly available COVID lesion segmentation AI model ^66^. Although these initial predictions may be considered as a suggestion from an expert, which is a typical workflow for many AI data annotation solutions, a second verification from another human expert would likely further improve the annotation quality.

Finally, the statistical consensus ranking algorithm over multiple tasks, although it overcomes the limitations of ranking based on single evaluation metrics, is computed only at the image level. The ranking does not provide a measurement of the algorithm on the lesion level, thus without consideration of each lesion’s clinical relevance. Such information, which was nor available in our data, could be important for clinical diagnosis and tracking of disease progress. It could also provide a more granular interpretation of the strengths and weaknesses of each algorithm, and a guidance on how to improve them.

## Conclusion

The COVID-19 Lung CT Lesion Segmentation Challenge - 2020 provided the platform to develop and evaluate AI algorithms for the detection and quantification of lung lesions from CT images. AI models help in the visualization and measurement of COVID specific lesions in the lungs of infected patients, potentially facilitating more timely and patient-specific medical interventions. Over one thousand teams registered to participate in the challenge participating in this challenge reflecting the engagement of the global scientific community to combat COVID-19. The AI models could be rapidly trained and showed good performance that was comparable to expert clinicians. However, robustness to “unseen” data decreased in the testing phase, indicating that larger and more diverse data may be beneficial for training. A more granular interpretation of the strengths and weaknesses of each algorithm might highlight pathways on the road towards a future where AI and deep learning might help standardize, quantify, assess disease response, select patients or therapies, or predict outcomes. But first steps first, as the scientific community builds multi-disciplinary teams to develop new tools and methodology to walk before we run. As more AI applications are being introduced in the biomedical space, it is essential to adequately validate and compare the functionality of these applications through challenges as proposed in this paper.

## METHODS

### Rank 1: “Semi-supervised Method for COVID-19 Lung CT Lesion Segmentation”

**Team:** Shishuai Hu, Jianpeng Zhang and Yong Xia

**Affiliation:** Northwestern Polytechnical University, China

**Abstract:** We noticed that the dataset provided in this challenge came from the TCIA database. Although the data in the TCIA database are not labeled for the COVID-19 Lung CT Lesion Segmentation task, they can be used as unlabeled data to improve the generalization ability of the segmentation model. To this end, we developed a simple but effective semi-supervised approach to utilize abundant unlabeled infected CT images. Specifically, we employ nnUNet as the backbone of the segmentation network and train it using labeled data at first. Next, we utilize the trained segmentation model to generate the pseudo lesion masks of both labeled and unlabeled infected CT images. Finally, a segmentation network can be trained in a fully supervised manner by feeding the data with generated pseudo labels. We validated our method on COVID-19 Lung CT Lesion Segmentation Challenge. Compared with the vanilla fully-supervised segmentation network, our approach can improve the Dice Similarity Coefficient by 4.27% (from 72.38% to 76.65%) on the training set (5-fold cross-validation).

### Rank 2: “nnU-Net for Covid Segmentation”

**Team:** Fabian Isensee, Peter M. Full, Michael Götz, Tobias Norajitra, Klaus H. Maier-Hein

**Affiliation:** Division of Medical Image Computing, German Cancer Research Center, Germany

**Abstract:** nnU-Net is a robust out-of-the-box segmentation tool that automatically configures itself for each dataset it is applied to. We use it as a framework to implement five 3D U-Net configurations:

1. a low resolution residual U-Net with extensive data augmentation and batch normalization (BN)
2. a high resolution U-Net with extensive data augmentation and instance normalization (IN)
3. a high resolution residual U-Net
4. a high resolution plain U-Net with extensive data augmentation and IN and
5. a high resolution plain U-Net with extensive data augmentation and BN.

High resolution U-Nets have a patch size of 28×256×256 voxels and operate on data resampled to a common voxel spacing of 5×0.74×0.74mm. The low resolution U-Net operates on 5×1.14×1.14mm with a patch size of 40×224×192. Each configuration is trained as a 5-fold cross-validation. Additionally, 5 random 80:20 data splits are trained for each configuration. We use the standard nnU-Net hyperparameters for training.

The configurations listed above were selected based on their cross-validation performance on the training set. We should note that none of these substantially outperformed the nnU-Net baseline. The best performing model was 2) with an average Dice score of 75.43 vs 74.41 for the 3d_fullres baseline.

The 10 models from the 5 configurations are all ensembled for the test set prediction (50 models) through softmax averaging. No post-processing is applied. We only use the data provided by the challenge.

### Rank 3: “Automated Ensemble Modeling for COVID-19 CT Lesion Segmentation”

**Team:** Claire Tang

**Affiliation:** Lynbrook High School, USA

**Abstract:** We developed an automated U-Net model training and optimization pipeline. Our pipeline includes the automated data preprocessing, automated U-Net model training with various data inputs and various loss functions, as well as the automated best combination for ensemble modeling. For data preprocessing, we create both 2D and 3D images. For 2D, we construct each CT slice as training data. For 3D, we construct both low-resolution images via down-sampling and full resolution images. Then, the whole training data is split into 5-fold training sets. For U-Net model training, we automatically train the following models: 2D U-Net using 2D images, 3D U-Net using both low resolution and full-resolution 3D images, 3D cascade U-Net which is first trained low-resolution U-Net on low-resolution 3D images and then uses its prediction to further train a full-resolution U-Net. For each U-Net model, we use the following three loss functions: DiceCE loss which combines region-based soft Dice loss and distributional-based cross-entropy loss, DiceTopK loss which combines soft Dice loss and TopK loss, DiceFocal loss which combines soft Dice loss and Focal loss. For ensemble modeling, we automatically evaluate the combination of our trained models by considering the combination of 2 to 4 models. The best model combination is then selected to test on validation and testing dataset. Our results show the best Dice Coefficient via cross-validation results on the training set is 0.7288. Our submitted validation results achieve Dice Coefficient 0.7363.

### Rank 4: “COVID-19–20 Lesion Segmentation Based on nnU-Net”

**Team:** Qinji Yu, Qikai Li, Kang Dang

**Affiliation:** Shanghai Jiao Tong University, China

**Abstract:** In the COVID-19 Lung CT Lesion Segmentation Challenge, we use nnU-Net which refers to a robust and self-adapting framework for medical image segmentation automatically. In view of the 3D CT data type in the challenge, we choose 3D U-Net to serve as the network architecture. Limited by the amount of available GPU memory, we try to train this architecture on 3D CT patches instead of the optimal whole CT scans. Firstly, we do preprocessing to all the training CT scans including cropping, resampling and normalization. After preprocessing, we divide the total 200 training CT scans into 5 folds randomly to perform 5-fold cross-validation on the training dataset and all models will be trained from scratch. The following augmentation techniques are applied on the fly during training: random rotations, random scaling, random elastic deformations, gamma correction augmentation and mirroring. We trained our networks with a noise-robust Dice loss for 400 epochs. During the inference stage, all inference is done patch-based. For the test cases we use the five networks obtained from our training set cross-validation as an ensemble to further increase the robustness of our models.

### Rank 5: “Leveraging state-of-the-art architectures by enriching training information - a case study”

**Team:** Jan Sölter (1), Daniele Proverbio (1), Mehri Baniasadi (1), Matias Nicolas Bossa (1), Vanja Vlasov (1), Beatriz Garcia Santa Cruz (2,1), Andreas Husch (1)

**Affiliation:** (1) Univ. of Luxembourg, Luxembourg Centre for Systems Biomedicine, Belvaux, Luxembourg, (2) Centre Hospitalier de Luxembourg, National Dept. of Neurosurgery, Luxembourg City, Luxembourg

**Abstract:** Our working hypothesis is that key factors in COVID-19 imaging are the available imaging data and their label noise and confounders, rather than network architectures per se. Thus, we applied existing state-of-the-art convolution neural network frameworks based on the U-Net architecture, namely nnU-Net [3], and focused on leveraging the available training data. We did not apply any pre-training nor modified the network architecture. First, we enriched training information by generating two additional labels for lung and body area. Lung labels were created with a public available lung segmentation network and weak body labels were generated by thresholding. Subsequently, we trained three different multi-class networks: 2-label (original background and lesion labels), 3-label (additional lung label) and 4-label (additional lung and body label). The 3-label obtained the best single network performance in internal cross-validation (Dice-Score 0.756) and on the leaderboard (Dice-Score 0.755, Haussdorff95-Score 57.5). To improve robustness, we created a weighted ensemble of all three models, with calibrated weights to optimise the ranking in Dice-Score. This ensemble achieved a slight performance gain in internal cross-validation (Dice-Score 0.760). On the validation set leaderboard, it improved our Dice-Score to 0.768 and Haussdorff95-Score to 54.8. It ranked 3rd in phase I according to mean Dice-Score. Adding unlabelled data from the public TCIA dataset in a student-teacher manner significantly improved our internal validation score (Dice-Score of 0.770). However, we noticed partial overlap between our additional training data (although not human-labelled) and final test data and therefore submitted the ensemble without additional data, to yield realistic assessments.

### Rank 6: “Ensembling 2D and 3D nnU-Net for fully-automated COVID-19–20 lesion segmentation”

**Team:** Tong Zheng, Luyang Zhang, Masahiro Oda, Kensaku Mori

**Affiliation:** Nagoya University, Japan

**Abstract:** Chest CT image processing for COVID-19 cases is becoming a big topic in the medical imaging field. Development and implementation of accurate COVID-19 CT image processing are fascinating challenges. In this COVID-19–20 lesion segmentation challenge, we used U-Net architecture as a baseline segmentation framework. The nnU-Net, which is based on U-Net is an image segmentation framework that automatically adapts its architectures to a given image geometry.

We trained 2D and 3D low-resolution nnU-Net on the training dataset (199 cases). Patch size for training 2D nnU-Net was 512×512 pixels (same as the resolution of each axial slice). Patch size for training 3D nnU-Net was 28×256×256 voxels (downsample each axial slice to ½ scale). The 2D nnU-Net was trained for 1,000 epochs, and 3D

nnU-Net was trained for 200 epochs. We used the Dice loss and the cross-entropy loss in the training process.

We merge 2D and 3D low-resolution nnU-Net’s outputs in the inference process. The trained 2D and 3D low-resolution nnU-Nets take the CT images as inputs. We obtain prediction results from softmax layers as outputs from 2D nnU-Net (result ***a***) and 3D low-resolution nnU-Net (result ***b***). Prediction results are the same size as the input image (512×512 pixels each slice). We calculate the mean of prediction results ***I*** = (***a***+***b***) / 2 for evaluation. Then we assign segmentation labels based on the prediction result I at each voxel. If the intensity of a specific voxel in ***I*** is larger than 0.5, we assign the foreground label (lesion) to such a pixel. At last, we also removed small connected components from the output. In the experimental results, we obtained a mean Dice coefficient of 0.7456 on the training dataset (2D nnU-Net, five-fold evaluation) and 0.6213 on the validation dataset (2D nnU-Net). On the test dataset (2D nnU-Net + low-resolution 3D nnU-Net), Dice coefficient score was 0.6392, and the mean Hausdorff95-Score was 118.6340.

### Rank 7: “Semi-3D CNN with ImageNet pretraining for segmentation of Covid lesions on CT”

**Team:** Vitali Liauchuk

**Affiliation:** United Institute of Informatics Problems (UIIP), Belarus

**Abstract:** The utilized network starts with 2D slice-wise convolutions and performs slice-wise extraction of a pyramid of features with use of an ImageNet-pretrained VGG16 model. Then a UNet-like decoder is attached to the feature pyramid. Indecoder, the convolutions are performed with 3D kernels. Max-pooling in the encoder and upsampling in the decoder are performed slice-wise in this version, though optionally can be made 3D as well.

The CNN training was performed with the use of the MONAI framework, the training parameters are mostly similar to the default ones. Data augmentation was extended by Gaussian blurring and sharpening and contrast adjustment. The probability of affine transform was increased to 0.3.

The training was performed at two stages:

1. Starting with ImageNet weights in the encoder and random weights in the decoder; learning rate: 0.0001; loss: Dice + 10 * Cross-Entropy; ~150 epochs.
2. Starting with the checkpoint with the highest Dice on validation subset; learning rate: 0.0001; loss: Dice; few epochs.

The Train dataset was split into training and validation subsets (“domestic”) in different ways. Depending on the split version the average Dice score on the domestic validation subset varied from 0.751 to 0.765 for the best runs. On the challenge Validation set, the best run resulted in 0.717.

The Test set submission was averaged over three trained models resulting from three different custom train/validation splits and resulted in 0.646 average Dice score.

### Rank 8: “3D Automated Chest CT Image Segmentation of COVID-19 with nnU-Net framework”

**Team:** Ziqi Zhou, Li Kang, Jianjun Huang

**Affiliation:** College of Electronics and Information Engineering, Shenzhen University, China

**Abstract:** Ground glass opacities in CT images are important indicators to diagnose COVID-19, and segmenting them is significant for diagnosis, treatment, and prognosis. In this paper, we propose a simple method based on nnU-Net training pipeline. First, we preprocess CT images using data augmentation to generate enough data for training, thus reducing the risk of overfitting. Then, we train the 3D high-resolution network and the 3D low-resolution network respectively with five-fold cross-validation. After the training, we select the best-performing network of each type for ensemble modeling. This is because we found through experiments that the ensemble of a few premium models is better than that of many mediocre models, since the former makes our model less affected by noise labels and causes false positives. Moreover, the ensemble of different resolutions can complement information at different semantic levels in images. This method uses neither pseudo-labels during the validation and test phases nor extra data. Dice coefficient of our model reaches 0.658 with all test cases, and particularly, 0.723 in the seen domain and 0.593 in the unseen domain.

### Rank 9: “Segmentation of COVID-19 lung lesions in CT using nnU-Net”

**Team:** Jan Hendrik Moltz, Alessa Hering, Hannah Strohm, Felix Thielke, Volker Dicken, Bianca Lassen-Schmidt

**Affiliation:** Fraunhofer Institute for Digital Medicine MEVIS, Germany

**Abstract:** We used the nnU-Net framework to train a convolutional neural network for segmenting COVID-19 lung lesions in CT in a fully automatic manner. We trained only the 3D U-Net in a single fold on the training data. We achieved a mean Dice coefficient of 0.793 on the training data and 0.744 on the validation data.

### Rank 10: “Automatic COVID-19 detection and segmentation from lung computed tomography (CT) images using 3D cascade U-net”

**Team:** Bruno Oliveira, MSc 1,2,3,4, Pedro Morais, PhD 1, Helena R. Torres, MSc 1,2,3,4, Fernando Veloso, MSc 1,2,3, Jaime C. Fonseca, PhD 4, João L. Vilaça, PhD 1

**Affiliation:** (1) 2Ai – School of Technology, IPCA, Barcelos, Portugal; (2) Life and Health Sciences Research Institute (ICVS), School of Medicine, University of Minho, Braga, Portugal; (3) ICVS/3B’s - PT Government Associate Laboratory, Braga/Guimarães, Portugal; (4) Algoritmi Center, School of Engineering, University of Minho, Guimarães, Portugal

**Abstract:** The early diagnosis of COVID-19 is fundamental for the patient treatment and management of the medical facilities. Thus, lung computed tomography (CT) images have been used to detect early indicators of COVID-19, namely ground-glass opacities. Owing to the large field of view of these images, automatic segmentation strategies are required, facilitating the clinical evaluation and speeding up the diagnosis of COVID-19. In medical imaging encoder-decoder, DCNNs have proved to be the best architectures for medical imaging segmentation. Here, we propose to use a coarse-to-fine 3D U-net approach. Firstly, the training images are downsampled and used to train a 3D low-resolution U-net. Next, the segmentation from the lower resolution training is used to crop the region-of-interest. The remaining volume is upsampled, and a new 3D U-net is further trained using the concatenation of high-resolution images with the coarse segmentation result. The two U-net were trained separately with the loss being defined as a combination of DICE and Cross Entropy. Finally, post-processing is applied to remove noncoherent anatomical results, namely lesions detected outside the lungs. Average Dice coefficients of 88.1% and 75.7%, average surface distances of 2.68 mm and 4.8 mm, and 95th quartile of Hausdorff distances of 9.91 mm and 78.3 mm were achieved for the training and validation dataset, respectively.

## Figures and Tables

**Table 1 |:** Top-10 finalists after statistical ranking. “Value” represents the average rank the algorithm achieved across all tasks. We also show if methods were automated, used external data for training, the input data dimensions used in the algorithms, and the network architecture.

Rank	Value	ID #	Fully Automated	Extra Data	Pretrained	Ensemble	Data Dimension	Network Architecture	Authors	Country
**1**	2.6	53	✓	✓	✗	✗	3D	nnU-Net	S. Hu et al.	China
**2**	6.0	38	✓	✗	✗	✓	3D	nnU-Net	F. Isensee et al.	Germany
**3**	7.7	65	✓	✗	✗	✓	2D/3D	nnU-Net	C. Tang	USA
**4**	8.4	58	✓	✗	✗	✓	3D	nnU-Net	Q. Yu et al.	China
**5**	8.5	31	✓	✗	✗	✓	3D	nnU-Net	J. Sölter et al.	Luxembourg
**6**	9.2	50	✓	✗	✗	✓	2D/3D	nnU-Net	T. Zheng & L. Zhang	Japan
**6**	9.2	68	✓	✗	✓	✗	2D/3D	VGG16 Hybrid, MONAI	V. Liauchuk et al.	Belarus
**8**	9.4	95	✓	✗	✗	✓	3D	nnU-Net	Z. Zhou et al.	China
**9**	10.6	29	✓	✗	✗	✗	3D	nnU-Net	J. Moltz et al.	Germany
**10**	11.3	15	✓	✗	✗	✗	3D	U-Net	B. Oliveira et al.	Portugal

**Table 2 |:** Dice coefficients of the top-10 algorithms on (left) all test data, (middle) “seen” data (Dataset 1), and (right) “unseen” test data (Dataset 2).

All test cases:					“Seen” test cases:					“Unseen” test cases:			
ID #	mean	std	median		ID #	mean	std	median		ID #	mean	std	median
53	0.666	0.236	0.754		38	0.740	0.195	0.797		53	0.598	0.264	0.700
58	0.658	0.242	0.741		53	0.734	0.182	0.782		95	0.593	0.258	0.677
95	0.658	0.237	0.729		31	0.729	0.190	0.769		58	0.588	0.263	0.724
38	0.654	0.268	0.763		65	0.729	0.186	0.778		15	0.581	0.264	0.670
15	0.649	0.242	0.716		58	0.728	0.195	0.789		68	0.570	0.276	0.703
68	0.646	0.251	0.753		95	0.723	0.193	0.783		38	0.569	0.302	0.729
31	0.645	0.265	0.753		68	0.723	0.196	0.779		50	0.562	0.279	0.692
65	0.644	0.258	0.754		29	0.722	0.187	0.711		31	0.561	0.300	0.685
50	0.639	0.252	0.733		15	0.717	0.197	0.751		65	0.559	0.291	0.686
29	0.634	0.259	0.705		50	0.716	0.194	0.773		29	0.545	0.289	0.647
